# Thermo-Priming Mediated Cellular Networks for Abiotic Stress Management in Plants

**DOI:** 10.3389/fpls.2022.866409

**Published:** 2022-05-13

**Authors:** Ambreen Khan, Varisha Khan, Khyati Pandey, Sudhir Kumar Sopory, Neeti Sanan-Mishra

**Affiliations:** Plant RNAi Biology Group, International Centre for Genetic Engineering and Biotechnology, New Delhi, India

**Keywords:** priming, high temperature stress, phytohormones, chromatin modification, molecular mechanisms

## Abstract

Plants can adapt to different environmental conditions and can survive even under very harsh conditions. They have developed elaborate networks of receptors and signaling components, which modulate their biochemistry and physiology by regulating the genetic information. Plants also have the abilities to transmit information between their different parts to ensure a holistic response to any adverse environmental challenge. One such phenomenon that has received greater attention in recent years is called stress priming. Any milder exposure to stress is used by plants to prime themselves by modifying various cellular and molecular parameters. These changes seem to stay as memory and prepare the plants to better tolerate subsequent exposure to severe stress. In this review, we have discussed the various ways in which plants can be primed and illustrate the biochemical and molecular changes, including chromatin modification leading to stress memory, with major focus on thermo-priming. Alteration in various hormones and their subsequent role during and after priming under various stress conditions imposed by changing climate conditions are also discussed.

## Introduction

Plants being sessile by nature are perpetually manifested with various adverse abiotic and biotic conditions. These unfavorable conditions usually occur together; concurrently or sequentially and negatively impact plant growth, ultimately leading to loss of crop yields (Pandey et al., 2017; Hilker and Schmülling, 2019). Plants possess efficient signaling networks and a sophisticated innate immune system, which enables them to endure a combination of diverse stresses. They seem to have well-defined mechanisms and strategies to remember the impeding stresses by inducing “stress memory” (Martinez-Medina et al., 2016; Hossain et al., 2020).

The concept of memory in relation to plant biology was first indicated by Charles Darwin while analyzing the Venus flytrap (*Dionaea muscipula*), for its ability to recognize and grasp insects (Darwin and Darwin, 1880). The term “memory” has been used to describe processes used for acquiring, storing, retaining and retrieving information. In humans and animals, it has been mainly linked with neuronal communications. In case of plants, however, “memory” has been used to describe three behaviors namely, keeping time, chemical talks within self and cross-talks with surroundings (Leopold, 2014).

The work on the retention of memory was demonstrated by Gagliano and her colleagues by using a potted touch-me-not plant (*Mimosa pudica*) (Gagliano et al., 2018). The beneficial adaptable behaviors in plants were considered to be similar to non-conscious, involuntary primitive qualities. Prof. Rainer Hedrich and his team elucidated that various action potentials elicited by an insect in insectivorous plants could be effectively mimicked by electric pulses. The pulses appraised and forewarned the plants with regards to the size of the prey and constitution of the nutrients (Böhm et al., 2016). These experiments demonstrated that plants could learn in terms of counting, memorizing and interpreting the action potentials evoked due to mechanical stimuli and further interpreting these stimuli to effectuate gene expression (Scherzer et al., 2017).

The ability of plants to retain memory in response to milder impact of various biotic and abiotic challenges is called stress priming. In this review we will discuss the biochemical and molecular events induced during stress priming, which may be the cause or effect for the retention of memory. This stress memory can even be passed on to the next generation (Bruce et al., 2007). These changes may eventually help the plants to ward off the negative impact of subsequent severe stress. The descriptions mainly include examples from thermo-priming but examples from other stress priming events are also discussed to better explain the cellular molecular mechanisms.

## Stress Priming

Stress can appear at any stage during the development of a plant and it affects essential processes like photosynthesis, membrane stability, gene expression, RNA splicing, protein synthesis, etc (Sah et al., 2016). When exposed to stress plants undergo several changes, which eventually allow them to tolerate stress and survive. When plants are subjected to a mild or sub-lethal stress treatment and permitted to regain, they form a “memory” which ensures that future stress stimuli are not as detrimental as the severe stress given at the first time, even in sensitive plants (Mittler et al., 2012; Crisp et al., 2016). The “memory” response forms the basis of the process of stress priming to facilitate protection of plants upon exposure to subsequent harsh stress. Stress priming is also referred to as stress hardening, stress training, or stress conditioning. Priming has attracted intensive research over the last decade as primed plants show better adaptability and improved stress tolerance. Recent research interests are now focused on understanding and developing stress priming as a viable technique for improving plant tolerance to diverse stresses (Balmer et al., 2015; Martinez-Medina et al., 2016; Xiao et al., 2017; Liu H. et al., 2021).

There are several reports to support that an earlier exposure to a modest high temperature stress (HTS) eliciting stimulus can prime a plant to acclimate to a further exposure of same or different stress (Table 1). Alexandrov (1956) first used the term “heat hardening” to describe this occurrence and he observed that hardening time and temperature are inversely proportional. Likewise, thermo-tolerance can be induced in plants after priming with other biotic or abiotic stress (Table 1). Plants can also be primed for a better response to unfavorable conditions by treatment with specific chemicals like salicylic acid (SA) or JA (Aranega-Bou et al., 2014; Conrath et al., 2015), herbivory and by colonization with microbes like rhizobacterium species (Conrath, 2011; Pieterse et al., 2014; Liu and Avramova, 2016).

**TABLE 1:** Thermo-priming for enhancing plant tolerance to high temperature and other abiotic stresses.

Elicitor	Priming treatment			Subsequent stress			Effect of priming	Reference
	Plant stage	Duration	Plant	Stress	Duration	Plant Stage		
**HTS tolerance through somatic thermo-priming**								
30–38°C	2 weeks	Gradually increasing (30–36°C for 45 min each; 38°C for 90 min)	*Oryza sativa*	45°C	90 min	2 weeks	Up regulation of HSFs, HSPs and TFs due to changes in miRNA profiles	Kushawaha et al., 2021
29–38°C	Anthesis	Gradual increase from 29 to 38°C during the 1st h; 38°C for 6 h		38°C	2 days	Anthesis	Up regulation of HSPs	Jagadish et al., 2010
37°C	1 month	1 h	*Rhododendron hainanense*	42/35°C*	1 h; 7 days	1 month	Accumulation of HSPs, RCA1, CPN60b and pTAC5	Wang X. et al., 2020
37°C	5 days	1.5 h	*Arabidopsis thaliana*	44°C	45 min	5 days	Increased abundance and activity of ROF1, HSP90.1 and HSFA2; Rapid accumulation of HSP21 regulated by plastid localized metalloprotease FtsH6	Sedaghatmehr et al., 2016; Thirumalaikumar et al., 2021
	21 days	2 h			2 h	21 days	CSN5A regulated expression of HTS memory genes, *APX2* and *HSP22*	Singh et al., 2021
	5 weeks	1 h		45°C	3 h	5 weeks	Higher activity of APX, POD1; up regulation of ABA and ACC in roots	Prerostova et al., 2020
33.5°C	12 days	3 h		45°C	1.5 h	12 days	Increased production of branched chain amino acids, RFOs, lipolysis products and tocopherols	Serrano et al., 2019
38°C	27 days		*Solanum lycopersicum*	38°C		30 days	Improved stress avoidance by increase in evaporation and decrease in leaf temperature	Zhou et al., 2020
35°C	3 months	1 h	*Achillea millefolium*	45°C	5 min	3 months	Improved photosynthesis and synthesis of secondary compounds with antioxidative characteristics	Liu B. et al., 2021
5°C higher than control	Stem elongation, booting and anthesis	5 days	*Triticum aestivum*		5 days	Grain filling	Enhanced activities of antioxidant enzymes, reduction in ROS and malondialdehyde production; increased photosynthesis, stomatal conductance and chlorophyll content	Fan et al., 2018
36/32°C*	Tillering	48 h		36/32°C*	7 days	Anthesis	Primed plants were more efficient in re-mobilizing dry matter than non-primed plants	Mendanha et al., 2018
40°C	Germinating seeds	4 h		35/27°C*	5 days	Post anthesis	Leaf photosynthesis and antioxidation capacities were improved due to up regulation of photosynthesis, antioxidation and HSPs	Zhang et al., 2016b
32/24°C*	2 leaf seedlings	2 days		35/27°C*	5 days	2 leaf seedlings	Maintenance of a better redox homeostasis due to improved antioxidant capacity	Wang et al., 2014a; Fan et al., 2018
**HTS tolerance through meiotic thermo-priming**								
32/28°C*	Pre anthesis (9 leaf)	Pre anthesis: 2 days	*Triticum aestivum*	T-gen 34/30°C*	10th day post anthesis	6 days	Higher activities of antioxidant enzymes, increased rate of photosynthesis and dry matter translocation; up regulated LSD1	Wang et al., 2016
34/30°C*	Post anthesis (10th day)	Post anthesis: 7 days						
30°C	10 days	2 weeks	*Arabidopsis thaliana*	Seeds collected			memory of attenuated immunity, early flowering and post-transcriptional gene silencing (PTGS) release.	Liu J. et al., 2019
40°C	12 days (seedlings); early rosette stage	Gradually increasing temperature over 7 h from 18°C to 40°C; 40°C for 2 h		T-gen till G4 40°C	72 h	12 days	Accelerated flowering; maternally and paternally inherited epigenetic changes induced by exposure to abiotic stress over multiple generations can lead to reversible, *trans*-generational phenotypic changes	Suter and Widmer, 2013
50°C	5 days	3 h per day for 5 days		T-gen 50°C	5 days, 3 h per day	5 days	Increased expression of HSFA2 and reduction in MSH2, ROS1 and several *SUVH* genes	Migicovsky et al., 2014
42°C	2 weeks	3 h per day for 7 days	*Brassica rapa*	T-gen 42°C	3 h per day for 7 days	2 weeks	Regulation of BR metabolism, H^+^ ATPase activity, signaling by IMPL1, mRNA decay activity by RRP41 and epigenetic regulation by FAS2	Byeon et al., 2019
**Tolerance to other biotic/abiotic stress(es) through thermo-priming**								
45°C	12 days (3 leaf stage)	3 h	*Oryza sativa*	Cadmium (5 μM)	3 h, 30/25°C*	3 leaf stage seedlings	Transient induction of H_2_O_2_ followed by increased activity of GR and APX	Hsu and Kao, 2007; Chou et al., 2012
35/29°C*	seeds of IRBB61	35/29°C*		biotic stress	48 h post inoculation	rice plant	Resistance to *Xanthomonas oryzae*, by suppressing ABA responsive genes	Cohen et al., 2017
35, 45, 50, and 55°C	3 leaf (seedlings)	30 min	*Hordeum vulgare*	Salt (200 mM)	30 min	3 leaf stage seedlings	Increased root growth, osmotic potential in leaves and gene expression (APX, CAT2, Cu/Zn SOD, BAS1, DRF1, MT2, NHX1, HSP17, HSP18 and HSP90)	Faralli et al., 2015
38°C	mature green plants	12 h	*Solanum lycopersicum*	Chilling (arginase inhibitor NOHA, 30 μM)	12 h	mature green plants	Increased production of proline and putrescine; Increased activities of arginase, SOD, APX and CAT leading to reduced oxidative damage	Zhang et al., 2013b
40.5°C	2–3 months (plants)	2 h		*Pseudoidium neolycopersici* infection	9 days post inoculation	seedlings	Suppression of pathogen *Pseudoidium* by activating JA, ABA and peroxidase activity	Nožková et al., 2018
42°C	7 days (seedlings)	5 h	*Brassica campestris*	Salt (150 mM NaCl) and drought (20% PEG 6000)	48 h	7 day (seedling)	Higher activities of APX, GPX, GR, GST, DHAR, CAT, Gly I, Gly II and lower levels of GSSG, H_2_O_2,_ MDA	Hossain et al., 2013b
42°C	Seedlings	4 h	*Zea mays*	Chilling (0.5°C), drought, salt (0.7 mol/L NaCl)	5 days	Seedlings	Induction of H_2_O_2_ production, diminished loss of coleoptiles vitality and reduced electrolyte leakage in primary roots	Gong et al., 2001
37°C	7 days (seedlings)	24 h	*Triticum aestivum*	Heavy metals (Al, Cd, Cu and Fe each at 0.02, 0.2, 2 and 20 mol m−^3^)	2 h	7 days	Possible role of glutathione, phytochelatins, HSPs	Orzech and Burke, 1988
24/18°C*	3–5 leaf seedlings	21–24°C day time, 15–18°C night time		biotic stress (Stripe rust)	18–20 days post inoculation	Seedlings	Warm air provided resistant to stripe rust. Identified QTL, QYrlo.wpg 2BS associated with HTSAP	Carter et al., 2009
38°C	Young plants (10 leaves)	10 h	*Vitis vinifera*	Chilling stress (0.5°C)	10 h; 20	Young plants (10 leaves)	Organelle ultrastructure maintenance, reduced lipid peroxidation and membrane leakage	Zhang et al., 2005
40°C	hypocotyl	4 h	*Vigna radiata*	Chilling stress (2.5°C)	6 days	Hypocotyl	Chilling resistance by preventing membrane leakage	Collins et al., 1995
40°C	Seedling	1 h	*Cucumis sativus*	Chilling stress (2.5°C)	96 h	Seedling	Chilling resistance by root elongation and ion leakage	Jennings and Saltveit, 1994
38°C	7 days cultured pith tissue	2 h	*Nicotiana tabacum*	Salinity stress (1.2 % NaCl)	3 h	Tobacco cells	H_2_O_2_ causes expression of CAT, SOD, APX, GR and MAPKs leading to salt tolerance	Harrington and Alm, 1988
38°C	7 days (seedlings)	1.5 h	*Arabidopsis thaliana*	Anoxia	28 h	Seedling	Production of HSPs induce anoxia tolerant mechanisms	Banti et al., 2008
**Thermo-tolerance through priming with other biotic/abiotic stress**								
Water stress (3.5L in pot)	fully expanded first leaf	14 days	*Phaseolus vulgaris*	38°C	38°C	Trifoliate leaf plant	Sustained lutein and xanthophyll pigments, enhanced PG concentration, reduced unsaturation of thylakoid lipids	González-Cruz and Pastenes, 2012
Drought stress	2 months	8 days	*Festuca arundinacea*	38/33°C*	25 days	2 months	Enhanced accumulation of phospholipids and glycolipids for membrane stabilization and stress signaling	Zhang et al., 2019b
Cold stress (8°C)	100 days berries after full bloom	3 h	*Vitis vinifera*	45°C	4 h	100 days berries	Regulates SA and phospholipase D; Reduces membrane permeability and MDA contents, increases HSP73	Wan et al., 2009
Cold stress (0°C)	Seedling	4 days	*Hordeum vulgare*	35°C	5 days	Seedling	Increased activities of antioxidant enzymes CAT, APX, GR and SOD	Mei and Song, 2010
Fungus *Paraphaeosphaeria quadriseptata*_25c.f.u	Seedling	4 days	*Arabidopsis thaliana*	45°C	75 min	Seedling	Fungal secondary metabolites MON and RAD can bind and inhibit plant HSP90. MON leads to expression of HSP101 and HSP70 to promote HTS tolerance	McLellan et al., 2007
Endophyte *Curvularia protuberata*	1 month	Symbiotic relationship	*Dichanthelium lanuginosum*	45–65°C	3–10 days	1 month	Fungal endophyte produces cell wall melanin that may dissipate heat along the hyphae and/or complex with ROS generated during HTS	Redman et al., 2002
**Thermo-tolerance through priming with metabolites**								
Proline and glycine betaine (20 mM)	Single noded buds	8 h	*Saccharum sp.*	42°C	5 h	Sprouting buds	Restricted H_2_O_2_ production, improved K^+^ and Ca^2+^ content, increased concentration of free sugars	Rasheed et al., 2011
Ascorbic acid (70 ppm) and Hydrogen peroxide (30 ppm)	Sowing and reproductive stages	24 h spray	cotton plant	45/30°C*, 38/24°C*, 32/20°C*	7 days	Reproductive stage	Increased chlorophyll content, photosynthesis, fiber quality, SOD and CAT activity, net photosynthetic rate, chlorophyll content, fiber quality	Sarwar et al., 2018
Trehalose (30 mM)	6 years (plants)	3 days treatment	*Paeonia lactiflora* Pall.	40°C	3 days	6 years (plants)	Osmotic protection, decreased MDA, H_2_O_2_ and relative electric conductivity	Zhao et al., 2019
Glutathione (0.5 mM)	6 days (seedlings)	24 h	*Vigna radiata*	42°C	48 h	Seedling stage	Enhanced antioxidant and glyoxalase activities	Nahar et al., 2015
Sodium hydrosulfide (0.5 mM)	2.5 days (seedlings)	12 h	*Zea mays*	47°C	15 h	Seedling stage	Accumulation of endogenous betaine by activating BADH	Li and Zhu, 2015
Spermidine (1.5 mM)	grain filling	3 to 5 DAP	*Oryza sativa*	40°C	5 days	Grain filling	Enhanced seed germination percentage, grain quality, seedling shoot height and antioxidant enzyme activity	Fu et al., 2019

*indicates day/night temperatures.

The effect of priming can be short-term or long-term and the process can be achieved by exposing plants at three different developmental stages to different abiotic and/or biotic elicitors:

(a) Priming of seeds helps to evade diverse stresses at the time of germination by inducing cross-stress tolerance. Seed priming accelerated germination and emergence to allow seedlings to escape abiotic stress such as HTS (Banerjee and Roychoudhury, 2020; Kumari et al., 2021).

(b) Priming at the post-embryonic stage increases the likelihood of the plant for surviving through the environmental changes. Its effects are mainly observed in the existing generation so it is also categorized as intergenerational, mitotic or somatic stress memory. Yucel et al. (1992) demonstrated that pre-heat treated wheat seedlings displayed thermal tolerance to HTS and showed reduced loss in photosynthetic capacity.

(c) Priming of parental population, such that the effects are transmitted to progenies constitute *trans*-generational priming. Its effects influence the future generations so it is also categorized as meiotic stress memory (Munné-Bosch and Alegre, 2013). In *trans*-generational stress priming, the memory for response to environmental stresses are likely to be packaged into seeds along with other resources and these provide crucial information to the new seedling during germination and beginning of plant development (Herman and Sultan, 2011). Studies in *Brassica rapa* indicated that changes in the profiles of non-coding RNA due to HTS could be transmitted to the next generation (Bilichak, 2015). The effect of HTS on seed germination and seed vigor in the progeny from thermo-primed parents has been studied in wheat (Liu, 2020; Zhou et al., 2020).

Initially it was proposed that memory of biotic priming is conferred *trans*-generationally to protect the progeny against recurring biotic stresses (Luna et al., 2012), but soon it was discovered that memory of abiotic stresses is also passed on to the next generations.

Priming can also be categorized based on the nature of the stress signals into cis- and *trans*- priming. When the eliciting stimulus and the response-triggering stress are of the same nature it is termed as “cis-priming.” If the eliciting stimulus is different from the response-triggering stress it is termed as “*trans*-priming” (Hilker et al., 2016).

### *Cis*-Priming

Priming with a particular biotic or abiotic stress can stimulate plant tolerance to subsequent occurrence of same stress and such examples are categorized as *cis*-priming. It was unequivocally demonstrated using different plants that priming with high temperatures could help in increasing thermo-tolerance to subsequent HTS (Wang et al., 2014a; Zhang et al., 2016b). The preservation of thermo-memory involved expression of specific genes and epigenetic markings (Shi et al., 2016). In wheat seedlings, thermo-priming for two consecutive days with temperature which was 8°C higher than day/night temperatures, increased the tolerance of mature plants to subsequent HTS occurring after anthesis. The primed plants displayed enhanced photosynthetic capacity, higher scavenging capacity of reactive oxygen species (ROS) and greater grain starch accumulation, as compared to plants, which were not primed (Wang X. et al., 2012). These traits were attributed to the up regulation of expression of antioxidant genes in the primed plants (Wang X. et al., 2012; Wang et al., 2014a). High temperature exposure to 10 days old *Arabidopsis thaliana* induced attenuated memory, early flowering and post-transcription gene silencing in the next generation (Suter and Widmer, 2013; Liu J. et al., 2019). Pre-exposure to sub-lethal high temperatures boosted thermo-tolerance of rice plants and their progenies at the vegetative stage (Shi et al., 2016).

Similarly, priming plants with low temperatures was shown to successfully boost their tolerance to cold stress (Li X. et al., 2014). Seven days pre-exposure of wheat plants at tillering stage to a temperature which was 5°C less than surrounding environmental temperature (10°C), could relieve the damaging impact of low temperature (14°C) stress (Li X. et al., 2014). Cold priming resulted in accumulation of metabolites like sucrose, proline and other osmolytes thereby contributing to the stability of cellular membranes against cold stress (Iba, 2002). It also induced the expression of genes encoding antioxidant enzymes, electron transport chain during photosynthesis, synthesis of chlorophyll and starch thereby ameliorating cell membrane damage and protecting photosynthesis apparatus (Byun et al., 2014; Li X. et al., 2014).

### *Trans*-Priming

*trans*-priming includes all cases where pre-exposure to a particular biotic or abiotic stress can stimulate plant tolerance to subsequent occurrence of another stress. For instance, drought priming wheat plants at the stem elongation stage induced cross-tolerance to HTS at grain filling stage and reduced yield loss (Wang et al., 2015). Drought primed plants exhibited increase in rate of carboxylation and photosynthesis and reduction in rate of energy loss under HTS (Wang et al., 2015). Both drought primed and unstressed control wheat plants showed similar levels of total protein, glutenin macropolymers and high molecular weight glutenin subunits, which suggested that drought priming protected the grain quality under HTS (Zhang et al., 2013a). Similarly, intermediate drought stress during early seedling stage was found to reduce the damage of subsequent cold stress in wheat by activation of antioxidant enzymes and maintenance of photosynthesis (Li X. et al., 2015). In wheat, pre-treatment of the parent plants with drought or HTS during grain filling imparted *trans*-generational cross-tolerance to their offspring against HTS at post-anthesis stage (Wang X. et al., 2018). The progenies showed higher grain yield, better maintenance of leaf photosynthesis, enhanced activities of antioxidant enzymes and reduced cell membrane damage (Wang et al., 2016). Drought primed plant progenies also showed cross tolerance to HTS during grain filling due to increased in rate of photosynthesis, reduction of membrane damage and increase in grain yield (Zhang et al., 2016a). The imprint of *trans*-priming of stress tolerance was also observed in rice, tobacco, radish and alfalfa (Bruce et al., 2007; Choi and Sano, 2007; Vu et al., 2015).

*Trans-*priming may be facilitated as similar or overlapping signaling pathways are involved in the perception and response to most abiotic stresses in plant (Zhu, 2016). HTS resulted in acquired resistance against the heavy metals such as cadmium, aluminum, iron and copper (Orzech and Burke, 1988). It was shown that HTS treatment increased the production of antioxidant enzymes in plants, which helped to regulate the ROS production triggered by metals (Kochhar and Kochhar, 2005; Hsu and Kao, 2007). Thermo-priming could enhance the preservation of many fruits, particularly those from tropical or subtropical locations, prior to exposure to low temperatures. Response to thermo-priming was associated with accumulation of heat shock proteins (HSPs: HSP70 and HSP79), small HSPs (sHSP22, sHSP18.1, sHSP18.2) increase in antioxidant enzymes (APX, catalase), maintenance of ultrastructure, reduction of membrane leakage and lipid peroxidation. These processes were also associated with providing chilling tolerance (Lurie and Klein, 1991; Jennings and Saltveit, 1994; Collins et al., 1995; Sabehat et al., 1996; Sato et al., 2001; Rozenzvieg et al., 2004; Zhang et al., 2005). A variety of plants could develop cross-tolerance to salt stress after being pre-exposed to HTS. The thermo-memory prior to salt stress caused an increase in H_2_O_2_, which resulted in enhanced survival and decreased damage when later exposed to NaCl (Gong et al., 2001). Heat pre-treatment was also shown to increase salinity tolerance by improving antioxidant activity and glyoxalase metabolism in mustard, tobacco and cashew plants (Harrington and Alm, 1988; Hossain et al., 2013b; Pérez-Salamó et al., 2014).

Priming seeds with hormone solutions, bacteria and chemicals is also known to substantially improve seedling emergence, seed germination and plant growth under HTS (Hasanuzzaman and Fotopoulos, 2019). Seed priming with combination of ascorbic acid and SA improved rice growth under HTS (Kata et al., 2014). Priming seeds with bacteria such as *Bacillus* spp. and *Azospirillum* spp. improved tolerance against HTS by reducing ROS production (Abd El-Daim et al., 2014). Wheat seeds inoculated with rhizobacteria substantially increased thermo-tolerance of the seedlings (Yang et al., 2009; Anderson and Habiger, 2012). The endophytic bacteria present inside plant tissues have also been implicated in priming process to promote plant growth (Singh et al., 2021). Recently it was reported that colonization by Enterobacter sp. SA187, isolated from root nodules of the desert plant *Indigofera argentea* lead to HTS tolerance in wheat seedlings under field conditions (Shekhawat et al., 2021). Similarly, endophytic fungus, *Paecilomyces formosus* (Waqas et al., 2015) and arbuscular mycorrhizal fungi (AMF) (Begum et al., 2019) were shown to improve plant growth under HTS. AMF improved growth by enhancing uptake of water and nutrients, increasing the activity of antioxidant enzymes and improving the rates of photosynthesis (Zhu et al., 2011; Maya and Matsubara, 2013).

Priming of plants by foliar spray of various micronutrients also enhanced their performance under HTS. The foliar spray of boron and iron was effective in attenuating both HTS and drought stress by securing high water content (Kumari et al., 2019). Selenium acts as an osmo-protectant by improving membrane permeability and activities of anti-oxidant system under HTS (Djanaguiraman et al., 2010). Exogenously applied proline protected carbon metabolism and antioxidant enzymes in chickpea and thereby provided better tolerance to HTS (Kaushal et al., 2011).

The integration of priming memory in plants with different molecular techniques may have a major impact on vigor. Although common stress response mechanisms have been shown to provide cross-tolerance to a variety of abiotic and biotic stresses, different plant species have varied abiotic stress sensitivities and the mechanisms used to protect against stress and the cross-talk between these stresses may be species dependent.

## Molecular Mechanisms Behind Thermo-Priming

The response of plants to stress depends on their ability to rapidly transmit the perceived signals for initiating appropriate physiological, biochemical and molecular adjustments (Hasanuzzaman et al., 2013; Kollist et al., 2019). The stress signals get amplified through various second messengers like calcium ions (Ca^2+^) and ROS to regulate physiological and developmental responses. This leads to rapid alterations in the genetic machinery, which is achieved through a network of transcription factors (TFs) and microRNAs (miRNAs) (Kinoshita and Seki, 2014).

**FIGURE 1:**
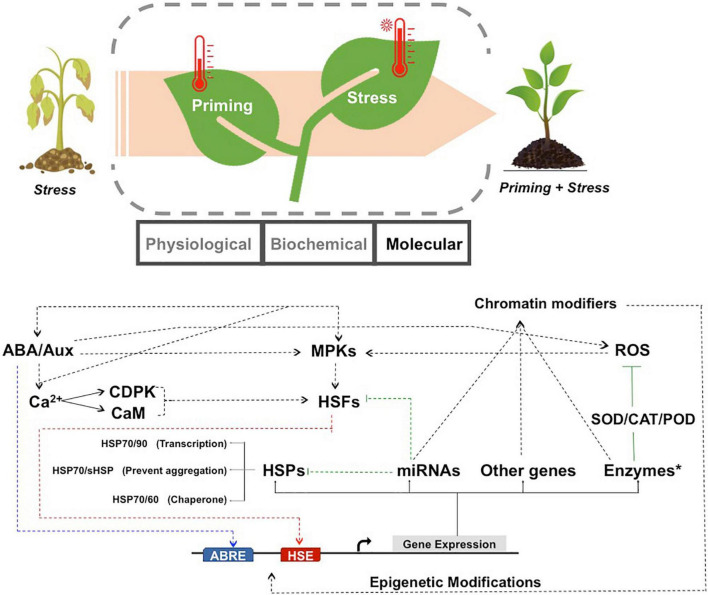
Schematic representation to show the molecular networks operative during thermo-priming in plants. Plants under HTS perform better when primed with a short exposure to high temperature. Priming leads to changes in the physiological, biochemical and molecular framework. The molecular mechanisms responsible for thermo-tolerance and HTS memory are indicated below. Temperature priming activates the hormonal networks and ROS, which transmit the signals through the Ca^2+^ dependent pathways and/or MPKs to regulate HTS-responsive gene expression. The Ca^2+^ ions can be conducted through the CDPK or CaM pathways to activate the transcription factors. The HSFs (red) and hormones responsive transcription factors (blue) bind to their respective cis-elements and promote the transcription of HSPs, antioxidant enzymes (like SOD, POD, CAT), miRNAs and other genes. HSPs inhibit thermal denaturation of cellular proteins through increased chaperone capacity (HSP70/60), prevention of aggregation (sHSP/HSP70) and increased transcription (HSP70/90). miRNAs negatively regulate their targets genes, some of which include HSPs, HSFs and chromatin modifiers to trigger positive or negative feedback loops for retention of high temperature memory. On exposure to stress, primed plants show enhanced thermo-tolerance due to preparedness of the molecular machinery. ROS, reactive oxygen species; HSP, heat shock protein; HSF, heat stress transcription factor; MPK, mitogen-activated protein kinases; CDPK, calcium-dependent protein kinase; CaM, Calmodulin; ABA, abscisic acid; Aux, auxin; SOD, superoxide dismutase; CAT, catalase; POD, Peroxidases; HSE, heat shock sequence element; ABRE, abscisic acid response elements.

The same molecular pathways also conferred memory during priming by triggering an early, appropriate and successful acclimation response (Bruce et al., 2007; Conrath, 2011; Walter et al., 2011; Kollist et al., 2019). The activation of basal cellular processes possibly ensured increased capacity to tolerate future stress and maintain complete fitness of the plant (Li S. et al., 2014). The hypothesis thus proposed, suggested that the rapid stress response phase in plants could serve as an intermediate stage in which generic morphological, biochemical and metabolic changes occurred to effectively counteract the detrimental effects of subsequent stress (Figure 1). These processes might involve some overlap with the responses activated during later stages of stress. Together they help in preventing irreversible damage and pre-inducing the stress response cascades. It is possible that a cellular feedback mechanism from the early stress response of plants provides memory by modifying or altering the later plant response during re-occurring stress events (Kollist et al., 2019).

There are now several reports from studies on thermo-priming and other stress priming events that point towards the underlying mechanisms. Analysis of transcriptome and proteome of heat primed plants showed that the translation of genes encoding cellular receptors, signal transducers, chaperones, enzymes associated with the process of photosynthesis and sucrose synthesis, transcription factors and HSPs were positively regulated whereas genes encoding enzymes involved in metabolism were negatively regulated (Xin et al., 2016; Zhang et al., 2016b; Xiao et al., 2017; Wang X. et al., 2020). The priming information was transmitted to the progenies in the form of cellular signals, HSPs and structural alterations in protein (Zhang et al., 2016b). Similarly, during excess light stress some transcripts were elevated within seconds and these were found to be essential for light stress acclimation (Suzuki et al., 2015). Priming with pathogens or biotic stress elicitors induced primary metabolism, pattern-recognition receptors, Mitogen-activated protein kinases (MAPKs) and chromatin modifications (Slaughter et al., 2012; Balmer et al., 2015; Conrath et al., 2015). Priming with mild drought or waterlogging in wheat seedlings induced proteins participating in oxidative stress, cell defense, carbon metabolism and photosynthesis (Xiao et al., 2017).

### Signaling Through Kinases

Synchronized phosphorylation and dephosphorylation of proteins play significant roles in priming plant tolerance to both abiotic and biotic stresses. The Receptor Like Kinases (RLKs) and MAPKs constitute two important signaling cascades for eliciting stress responses (Nakagami et al., 2005).

Receptor like kinases make-up one of the most extensive gene families found in plants and include a large subfamily of Leucine-Rich Repeats (LRR) that play essential roles in plant adaptation to diverse stresses (Monaghan and Zipfel, 2012). GbRLK, identified from *Gossypium barbadense* (cotton), plays a fundamental role in preventing wilt induced by *Verticillium dahliae* and tolerance to salinity and drought by regulation of stress-responsive genes (Zhao et al., 2013). Not much is, however, known about their role in HTS and thermo-priming responses as temperature-specific sensors or receptors are not yet known in plants (Liu J. et al., 2015). It is hypothesized that temperature-induced changes in membrane rigidity/fluidity might be involved in the perception of the temperature extremes (Guo et al., 2016).

Mitogen-activated protein kinases represent another large and conserved cascade of three sequentially functioning threonine and tyrosine (TXY) kinases, which mainly act downstream to the RLKs in the signaling events (Jonak et al., 2002; Sharma et al., 2020). They play a major role in amplification and transduction of intracellular signals. The MAPK (MPK) is the last enzyme in the cascade. It is phosphorylated and activated by its upstream MAPK kinase (MAPKK, MKK, or MEK) (Conrath, 2011). MAPKK activity is regulated by phosphorylation by the MAPKK kinase (MAPKKK or MEKK), which either directly or indirectly receives the stress signals from receptors or sensors (Takahashi et al., 2007). MAPKs can activate a variety of downstream effectors and TFs to control thousands of genes to ensure proper cellular functions. The involvement of MAPKs in HTS response has been experimentally validated (Sinha et al., 2011).

MPKs, MEKs, and MEKKs are considered as excellent candidates for transducing signals that mediate thermo-priming (Conrath, 2011). In wheat plants, genes encoding MAPK cascade, Ca^2+^ signaling kinases and other protein kinases were found to be up regulated in primed plants as compared to non-primed plants (Wang M. et al., 2016). In alfalfa, MKK1 and MKK4, which are intermediaries in wound-responsive MAP kinase signaling were also associated with priming for abiotic stress (Wassie et al., 2020). Transcriptome analysis also indicated a role for genes encoding Ca^2+^-dependent protein kinases and MAPKK in cold priming response in Arabidopsis (Byun et al., 2014). The accumulation of MPK3 and MPK6 was crucial for priming Arabidopsis plants and was related to increased *pal1* and *pr1* gene activation, ROS homeostasis, cold stress regulation and pathogen signaling (Beckers et al., 2009). Treatment with benzo (1,2,3) thiadiazole-7-carbothioic acid S-methyl ester (BTH), a synthetic equivalent of the SA resulted in the accumulation of transcript for *mpk3* and *mpk6*, as well as the gradual accumulation of MPK3 and MPK6 proteins (Beckers et al., 2009).

Forgetter2 (FGT2), a type 2C protein phosphatase, also plays an important role in HTS memory by maintaining the dynamics of cellular membranes (Castellanos et al., 2020). FGT2 also interacts with Phospholipase D (PLDα2) to produce phosphatidic acid (PA) and trigger lipid signaling for the maintenance of HTS memory. Mutants of *fgt2* and *pldα2* were defective in thermo-memory when exposed to reoccurring HTS.

In Arabidopsis, suppression of Target of Rapamycin (TOR) kinase is also well known for providing tolerance to different stresses (Bakshi et al., 2017; Wang P. et al., 2018; Dong et al., 2019). TOR overexpression lines were more immune to abiotic stress (Bakshi et al., 2017) but showed higher susceptibility to bacterial and fungal pathogens (De Vleesschauwer et al., 2018). TOR closely interacted with SnRK1 (Sucrose non-fermenting [SNF] related kinase 1) to regulate plant development and growth (Wang P. et al., 2018). It was demonstrated that in unstressed plants, TOR phosphorylated the ABA receptor to repress the ABA signaling pathway and prevent activation of stress responses. Whereas, ABA-dependent repression of TOR and SnRK1 supported plant survival under extreme hypoxia, starvation and darkness (Baena-González and Sheen, 2008). The SnRK2 isoform, SnRK2.10, is implicated in osmotic stress response independent of ABA. SNRK2.10 mutant plants showed increased sensitivity to dehydration stress with respect to wild type plants. The target proteins of this kinase included dehydrins, ERD10 and ERD14, which are also induced during salinity stress (Maszkowska et al., 2019).

Ca^2+^-sensing protein kinases (CPKS) or kinases interacting with Ca^2+^ sensors (CIPKs) and Ca^2+^ dependent protein kinases (CDPKs) are also associated with conferring stress resistance (Drerup et al., 2013; Mittler and Blumwald, 2015; Kudla et al., 2018). It was shown that exposure of apple trees to salt stress induced MdCIPK13, which phosphorylated to activate a sugar transporter, MdSUD2.2. The resulting sucrose accumulation provided the plants with protection from desiccation by increasing the osmotic pressure (Ma et al., 2019). The CDPKs take up a “primed conformation” as a first response to priming stimulus but their complete activation is possible upon exposure to subsequent harsh stress (Hake and Romeis, 2019).

### Reactive Oxygen Species Pathway

Superoxide (O^2–^), hydrogen peroxide (H_2_O_2_) and hydroxyl radicals (OH^–^) are the most prominent ROS in early reaction of plants to many types of stresses. The burst of ROS production under abiotic stresses is usually analogous with the depression of photosynthesis (Kurepin et al., 2015). Plants have both enzyme dependent and non-enzyme dependent pathways for scavenging and detoxifying ROS. The common ROS-scavenging enzymes are superoxide dismutase (SOD), ascorbate peroxidase (APX), catalase (CAT), glutathione peroxidase (GPX), peroxiredoxin (PrxR) and glutathione reductase (GR). The major non-enzymatic antioxidants are ascorbic acid (ASA), reduced glutathione (GSH), carotenoids and flavonoids (Mittler et al., 2004).

Thermo-primed wheat plants produced significantly less amount of O^2–^ as compared to non-primed plants (Wang et al., 2014a). Low ROS rates are generally regarded as necessary secondary messengers for cellular metabolism. Accumulation of ROS likely has diverse functions in phytohormone biosynthesis, transport and signaling so it may affect the plant responses mediated by the hormones during priming (Xia et al., 2015). It also plays an important role in cross-tolerance to both abiotic and biotic stress by eliciting the respiratory burst oxidase homologs (RBOHs) and regulation of ABA signals (Xia et al., 2014). However, high ROS levels result in lipid peroxidation, degradation of pigments, DNA damage and carbohydrate oxidation eventually resulting in programmed cell death (Suzuki et al., 2012). Cold priming and drought priming were shown to individually increase SOD, APX and GR activities to shield photosynthetic machinery and protect the cellular membranes (Thomashow, 1999; Wang et al., 2014b).

H_2_O_2_ can act as a second messenger and play diverse roles in stress priming and cross-tolerance to multiple abiotic stressors (Suzuki et al., 2012; Hossain et al., 2015; Mittler and Blumwald, 2015). The ABA-dependent accumulation of H_2_O_2_ could stimulate the expression of *cat1* gene (Guan et al., 2000). Earlier investigations have demonstrated that exogenous application of 10 mM H_2_O_2_ solution could prime maize leaves to confer tolerance to salt stress by modulating various cellular processes associated with high antioxidant enzyme activity and increasing content of soluble proteins and carbohydrates (Gondim et al., 2013). Priming with H_2_O_2_ improved photosynthesis in leaves exposed to HTS in cucumber and salt stress in maize by enhancing the activity of carbon fixation enzyme, protection of chloroplasts and modulation of metabolites (Khan et al., 2018; dos Santos Araújo et al., 2021). It was shown that plants treated with brassinosteroid (BR) exhibited increased NADPH oxidase activity and enhanced the levels of H_2_O_2_ in cucumber apoplasts. These plants also showed resistance to CMV and tolerance to photo-oxidation and low temperature stress (Xia et al., 2009).

The ROS pathway interacts with Methylglyoxal (MG), a potentially toxic compound for plant cells that is produced rapidly as a result of abiotic stress. MG is primarily a by-product of glycolysis but it can also be produced during photosynthesis and metabolism of protein and lipids (Hoque et al., 2016). Excessive cellular accumulation of MG leads to protein glycation resulting in their degradation, endoreduplication and increased DNA strand breaks, sister chromatic exchange and point mutations (Kaur et al., 2014; Hoque et al., 2016). It has been shown that the application of exogenous GSH reduces MG levels via the glyoxalase pathway (Yadav et al., 2005). At low concentrations, MG was found to function as a signal molecule in plants (Li, 2016) and it was associated with the regulation of stomatal movement. MG can cross-talk with ROS and Ca^2+^ to activate MAPK and regulate the expression of stress tolerance-associated genes, *rd29b* and *rab18* in an ABA-dependent manner (Hoque et al., 2012, 2016). Exogenous application of MG could improve wheat seed germination and seedling establishment under salt and cadmium stresses (Li, 2016; Li X. D. et al., 2017; Li et al., 2017a,b). Recently, MG was reported to act as a HTS priming agent in maize seedlings by inducing AsA-GSH cycle and the ROS-scavenging system (Majláth et al., 2020).

Co-activation of the glyoxalase and ROS-antioxidant pathways is an important feature of abiotic stress and cross-stress tolerances in plants (Yadav et al., 2005; Upadhyaya et al., 2011). It was observed that a short spell of cold shock (6°C, 5.5 h) or heat shock (42°C, 5 h) promoted cross-tolerance to drought and salt stresses in mustard (*Brassica campestris L*.) seedlings, due to the induction of cellular detoxification of ROS and MG (Hossain et al., 2013a,b). The thermo-priming response involved both ROS and MG pathways, which were implicated in promoting cross-tolerance to HTS, cold stress, drought stress, salt stress and heavy metals in rice (Hsu and Kao, 2007), pea (Streb et al., 2008), maize (Gong et al., 2001), wheat (Lei et al., 2005), tomato (Zhang et al., 2013b), and cucumber (Kang and Saltveit, 2001).

The abiotic stresses also triggered the formation of nitric oxide (NO), which disturbed the redox potential of the cell by affecting the antioxidant and MG, detoxification systems. NO is also involved in the post-translational S-nitrosylation and nitration of target proteins. The exogenous application of NO and hydrogen sulfide (H_2_S) could induce a primed state which protected plants from the negative effects of salt stress (Antoniou et al., 2020) and low-temperature stress (Amooaghaie and Nikzad, 2013) but their role in thermo-priming has not been elucidated so far. The molecular oxygen and/or O^2–^ anion interact with NO to yield reactive nitrogen species (RNS). These molecules act together to transduce signals during stressed conditions and maintain redox equilibrium inside the cell (Suzuki et al., 2012). The redox hub formed by interaction between ROS, RNS and reactive sulphur species (RSS) is referred to as RONSS (reactive oxygen, nitrogen and sulfur species) and is considered essential for priming strategies to enhance cross-tolerance (Terrile et al., 2020). The RONSS primed state induced translation of a pool of transcripts inclusive of those coding for antioxidant enzymes, osmo-protectant proteins and polyamine biosynthesis.

### Chaperons and Prions

One of the earliest indicators of the HTS response is the formation of heat shock proteins (HSPs). The HSPs act as molecular chaperones and are important for maintaining the structure and function of proteins, removing potentially toxic polypeptides and restoring protein homeostasis (Kotak et al., 2007; Timperio et al., 2008). They prevent the aggregation of non-native proteins by facilitating their refolding into their native conformation thereby minimizing toxicity of unfolded or denatured proteins during stress.

The HSPs are also associated with the maintenance of acquired thermo-tolerance (Xue et al., 2014). They are classified according to their molecular weight and functions into five broad families, namely HSP100, HSP90, HSP70, HSP60 and small HSP (sHSPs). The transcription of HSPs is stringently controlled by different members of HTS transcription factors (HSFs). The HSP90 and HSP70 represent the major families of HSPs and their activities are modulated by stress-induced HOP (Hsp70-Hsp90 organizing protein) protein (Carrigan et al., 2006). HSPs and HOP proteins were highly expressed in drought or thermo-primed plants, which helped in improving grain filling during HTS in wheat (Wang et al., 2014b; Zhang et al., 2016b) and rice (Kushawaha et al., 2021).

Under normal conditions, the HSP90.1 bound with ROF1 (rotamase FKBP 1), a member of the FKBP family, to form a cytoplasmic protein complex. Upon exposure to HTS, HSFA2 binds to HSP90.1-ROF1 and the resulting complex translocates to the nucleus, where it putatively functions to enhance the transcriptional activity of HSFA2 and maintain the expression of *hsp* genes regulated by it during HTS recovery (Meiri and Breiman, 2009). Accordingly, Arabidopsis plants overexpressing *rof1* displayed improved HTS memory while *rof1* mutant plants had impaired memory response. It was recently demonstrated that during the recovery phase NBR1 (Next to BRCA1) interacts with HSP90.1-ROF1 and facilitates their breakdown by autophagy to repress the response to HTS. Thus, HSP90 has a vital function in controlling of recovery from HTS and resetting the cellular memory of HTS in Arabidopsis. Loss-of-function mutants of NBR1 showed a stronger HTS memory (Thirumalaikumar et al., 2021). The HSP90 also displays hormonal cross talk by inducing TFs like ARFs and reducing the accumulation of transcriptional repressors like Aux/IAAs concentration (Wang R. et al., 2016; Watanabe et al., 2016). Studies in Arabidopsis and rice have indicated that HSP101-HSA32 interaction provides another conserved positive feedback loop during thermo-memory. Continuous accumulation of HSP101 during thermo-memory phase results in high abundance of HSA32. The HSFA32 in turn increases stability of HSP101 by retarding its degradation.

Several small HSPs like sHSP17.6II, sHSP22, sHSP18.2 are also associated with HTS memory. The Arabidopsis Hikeshi-Like Protein1 (HLP1) interacts with HSP70 to provide thermo-tolerance in plants (Koizumi et al., 2014). Induction of *Heat Stress Associated 32* (*hsa32*) gene by HSP101 is necessary for thermo-tolerance and HTS memory preservation (Wu et al., 2013). HSA32 works in tandem with Brushy1/Tonsoku/Mgoun3 (BRU1/TSK/MGO3) which is necessary for the sustained activation of HTS memory genes by inducing changes in chromatin structure (Suzuki et al., 2004; Takeda et al., 2004; Brzezinka et al., 2019).

Another interesting mode of biochemically reproducible memory is provided by sustainable changes in the protein conformation and function of prion domain proteins (PrD). These proteins are ubiquitously present in fungi, mammals and plants (Shorter and Lindquist, 2005; Chakrabortee et al., 2016). The PrDs were shown to switch between non-aggregated states and higher-order functional oligomers. The highly ordered aggregates acted as self-replicating entities that had the ability to propagate and could be transmitted to next generations. These characteristics enabled them to bestow stable alterations in biological states, which is important in molecular biology (Newby and Lindquist, 2013), but their significance in plant stress and memory remains largely unknown. A recent report has identified candidate PrDs in approximately 500 plant proteins using computational algorithms and predicted their diverse functional roles in stress and developmental responses including flowering time and thermo-sensory responsiveness (Garai et al., 2021). Transition from vegetative to reproductive stage also involves memorizing and integrating previously encountered environmental conditions. It was reported that Luminidependens (LD) proteins that are involved in regulating the timing of flowering in Arabidopsis behaved as prion-like conformational switch (Lee et al., 1994; Chakrabortee et al., 2016). The LD may be responsible for memorizing and assimilating the ambient signals required to control flower timing. Recently, an Arabidopsis PrD, ELF3, was found to be associated with thermo-sensory response (Jung et al., 2020).

## Regulation of Gene Expression

Response to stress results in alterations in gene expression and the foremost amongst them involve activation of transcription, stabilization of mRNA and synthesis of new proteins. The genes can be grouped to differentiate those regulating the “non-memory” response from the group of genes that delineate the “transcriptional memory” (Ding et al., 2013). This became evident from whole-genome transcriptome studies in Arabidopsis plants exposed to dehydration stress. Single exposure to stress identified >6500 differentially expressed genes, while upon repeated exposure, 4500 genes responded to each stimulus in a same way while 1963 genes generated considerably varied levels of transcripts each time.

Analysis of transcriptional changes, in *Arabidopsis thaliana* and *Zea mays* plants, in response to multiple stimuli indicated that transcriptional memory was based on an evolutionarily conserved mechanism that could discriminate between single and repeated stresses. The memory genes could induce or alter mRNA synthesis resulting in cellular changes and/or interactions between overlapping signaling pathways (Ding et al., 2014). These genes produced enzymes, osmolytes, dehydrins and chaperones, which were involved in cellular detoxification, protection and damage-repair. In Arabidopsis, stimuli like touch, wind, wounding, rain or transition of plants from light into darkness caused the translation of *tch* (touch induced) genes (Chehab et al., 2012). One of the members of this gene family was identified as a calmodulin homolog that could be involved in Ca^2+^ signaling (Chehab et al., 2009).

### Transcription Factors

Transcriptome studies on stress challenged plant tissues identified changes in the transcripts coding for different TFs. Several genes from the MYB, NAC, HSFs, WRKY ethylene response factors (ERFs or AP2), and zinc finger (ZNF) super families have been shown to provide cross-tolerance to abiotic and biotic stressors in plants.

The responses to HTS and thermo-priming are transduced through the MAP kinase pathway to induce the translation of HSFs, which are prime regulators of plant response (Zhang et al., 2016b). HSFs are a conserved family of TFs that bind directly to heat shock elements (HSEs) to control HTS-related gene expression (Ohama et al., 2017). The HSEs consist of tandem inverted repeats of the pentameric consensus sequence nGAAn (nTTCnnGAAnnTTCn) and the AGGGG motifs (Swindell et al., 2007; von Koskull-Döring et al., 2007). Variable number of HSFs are observed in plants, *viz.* rice (25 members), wheat (56 members), Arabidopsis (21 members), soybean (52 members), tomato (26 members), maize (30 members), carrot (35 members), pepper (25 members), cotton (40 members) etc. (Scharf et al., 2012; Xue et al., 2014; Guo et al., 2016; Yang X. et al., 2016; Li et al., 2019). HSFs are divided into three structural classes: A, B and C, with class A serving as the principal activator of thermo-tolerance genes (Guo et al., 2008; Scharf et al., 2012).

Among the Class A HSF, the HSFA1 subfamily participates as early response genes for activating the other HTS response factors, for instance HSFA2 and DREB2A (Guo et al., 2008; Liu et al., 2011a; Scharf et al., 2012). Recent investigations have shown that HSFA1a directly senses HTS and gets activated through modifications in its redox state (Liu et al., 2013). HSFA1a is involved in the expression of genes coding for HLP1, dehydration-responsive element-binding protein 2a (DREB2a), HSFA7a, HSFBs, HSFA2 and multi-protein binding factor 1C (MBF1C) by binding to HSEs present in their upstream regions. HSFA2 is highly stimulated during HTS and is crucial for growth as well as maintaining the thermo-tolerance response during recovery period. It is regulated by three other TFs, HSFA1b, HSFA1d and HSFA1e (Yeh et al., 2012; Li X. D. et al., 2017). Thermo-priming-induced HSFA2 transiently binds at the promoter region of HTS memory genes, thereby facilitating di- and tri-methylation of histone H3 (Lämke et al., 2016). This chromatin modification triggers hyper-activation of transcription at these loci under subsequent HTS. HSFA3 expression is regulated by DREB2a and DREB2c and is essential for thermo-tolerance (Yoshida et al., 2011; Sato et al., 2014). In tomato, HsfA3 shows strong phosphorylation by HTS activated MAP kinases (Sinha et al., 2011). Other HSFs like HSFA4a and HSFA8 are also involved in sensing the ROS and induction of antioxidant system in plants (Qu et al., 2013). HSFA9 is exclusively expressed in late seed development stages.

The Class B HSFs, such as HSFB1 and HSFB2b primarily act as transcriptional repressors to inhibit HTS-induced gene expression (Ikeda et al., 2011). They act as co-activator in combination with class-A HSFs (Ikeda et al., 2011; Fragkostefanakis et al., 2015). The role of Class C HSFs in HTS is not clear. TF-HSFC1a was found to be negatively controlled by early HTS treatment in rice (Mittal et al., 2009), while overexpression of HSFC2a-B in wheat increased the expression of heat associated genes, resulting in improved thermo-tolerance (Hu et al., 2018).

NAC binding sites are known to be present in the promoters of several HSFs e.g., HSFA1b, HSFA6b, HSFA7a, and HSFC1 (Guo et al., 2015). The NAC TF family is found in plants and has been linked to the regulation of development and a stress related response. Overexpression of OsNAC6 improved tolerance of rice plants to drought and salt by activation of many stress-responsive genes (Nakashima et al., 2007). Heat priming elevated the expression of ATAF1 but its level again decreased during thermo recovery phase (Alshareef et al., 2019). JUB1 and ANAC019, members of NAC-TF family, were reported to regulate HTS and heat memory. JUB1 was reported to show thermo-memory related expression, which was similar to that of HSFA2 and HSFA32 (Shahnejat-Bushehri et al., 2012).

The stress induced increase in ABA, signaled the activation of R2R3-MYB TF, TaPIMP1 in wheat. This increased resistance to the fungus, *Bipolaris sorokiniana* and tolerance to drought stress (Zhang et al., 2012). The MYB-TFs also conferred cross tolerance to abiotic stimuli such as salinity and drought in transgenic tobacco by increasing the activity of phenylalanine ammonia lyase (PAL) and SOD (Liu et al., 2011b).

The expressions of ERF/AP2-TFs and ZNF proteins were induced in cold-primed plants indicating their crucial roles in priming plant response to cold tolerance (Byun et al., 2014). In cereals, long-term exposure to cold lead to the stable induction of VRN1, a TF related to AP1 (Trevaskis et al., 2003; Yan et al., 2003). This process was accompanied by accumulation of H3K4me at the VRN1 locus (Huan et al., 2018) indicating a possibility of analogous pathway for regulating chromatin in long-term memory of cold in cereals.

Priming by dehydration induced ABA-dependent memory through recruitment of MYC2-TFs, mobilization of mediator complex and accumulation of Ser5P RNA Pol II (Liu and Avramova, 2016). The ABA-dependent expression of a stress inducible OsWRKY45 gene increased tolerance of Arabidopsis to salt and drought stress (Qiu and Yu, 2009). WRKY40 and Zat12, TFs showed a triphasic transient expression pattern during the response to stress. Their expression first peaked as a rapid response within a few seconds of stress (Suzuki et al., 2015), second time after a few minutes and third time after a few hours (Van Aken et al., 2016), indicating that TF pulses control the transcriptome in plants (Davletova et al., 2005).

### MicroRNAs

Genome-wide transcriptome and small RNA analysis has linked the non-coding RNAs to stress response in plants. The stress responsive expressions of several conserved and novel miRNAs have been captured in a variety of plants like rice, tomato, populus, mustard, Arabidopsis, wheat, switch grass, cavendish banana and french beans (Chen L. et al., 2012; Yu et al., 2012; Kruszka et al., 2014; Raghuram et al., 2014; Jyothi and Rai, 2015; Liu W. et al., 2015; Hivrale et al., 2016; Pan et al., 2017). Different miRNAs such a miR156, miR159 and miR160 have been found to play essential roles in response to HTS (Stief et al., 2014). Many miRNAs induce response to HTS and thermo-memory by regulating key TFs and enzymes. For instance, miR159 acts on the *GAMYB* transcripts, miR396 regulates transcripts of *WRKY* TFs, miR528 regulates transcripts encoding F-box protein and miR5054 acts on *cat* transcripts (Giacomelli et al., 2012; Wang Y. et al., 2012). The miRNA168 acts in thermo-tolerance memory by regulating the function of AGO1 in thermo-memory, which generates a negative feedback loop and changes the expression of a larger number of miRNAs (Vaucheret et al., 2006).

It has been shown that miR156 was highly induced after HTS, to down regulate the *Squamosa Promoter Binding Protein-Like* (*spl*) TFs. Thermo-priming seems to generate HTS memory in Arabidopsis by repressing the transcription of *spl2* and *spl11* transcripts by inducing transcription of miR156 (Yu et al., 2012; Stief et al., 2014). During recovery from HTS, the *spl* levels were restored. The HTS also induced a positive feedback loop consisting of HSFA regulated miR398. The levels of miR398 were increased in response to HTS for down regulating the *copper/zinc superoxide dismutase* gene (Guan et al., 2013). This lead to increased levels of ROS, which in turn induced the HSFs and HSPs.

Another study showed that miR167 regulated DNA methylation process in response to HTS (Wu et al., 2010; Sanchez and Paszkowski, 2014; Naydenov et al., 2015), indicating a possible role for imprinting in HTS memory. The expression of osa-miR531 is regulated under different abiotic stress conditions including short duration HTS by signaling through MAPKs (Raghuram et al., 2014). HTS was shown to enhance the synthesis of vitamin E in chloroplasts, which up regulated miR398 biogenesis by retrograde signaling and provided protection to the Arabidopsis plants (Fang et al., 2019). A role for tRNA-derived siRNA fragments in *trans*-generational transmission of thermo-priming memory has recently been reported in durum wheat (Liu, 2020).

Our group has shown that miR169:NFY module may play a crucial role in integrating stress memory induced during HTS priming with light regulated development (Khan et al., 2017; Kushawaha et al., 2019). NGS analysis of rice transcriptome showed that levels of several miRNAs, HSPs, and HSFs were dis-regulated by thermo-priming (Kushawaha et al., 2021). Several heat responsive miRNAs like osa-miR531a, osa-miR5149, osa-miR168a-5p, osa-miR1846d-5p, osa-miR5077, osa-miR156b-3p, osa-miR167e-3p were identified that acted on respective HSF or HSP targets to differentially alter gene expression (Kushawaha et al., 2021). The other differentially expressed transcripts were mainly associated with redox pathway, protein phosphorylations and regulation of transcription.

## Role of Chromatin Modification in Stress Priming

Epigenetic regulation resulting in chromatin modification is a common mechanism used to regulate transcriptional activity in eukaryotes. Chromatin is composed of negatively charged DNA associated with positively charged histones that are arranged as nucleosomes. Each nucleosome consists of two molecules of each four histones (H2A, H2B, H3 and H4) forming an octamer, which is wrapped by DNA. The nucleosomes are connected to each other by a DNA strand linked with histone H1 (Kornberg, 1974; Thoma et al., 1979).

The DNA and histones are subjected to covalent modifications such as methylation, acetylation, ubiquitination and poly-ADP ribosylation during gene regulation. DNA methylation and histone modifications are two main mediators of epigenetic regulation. DNA methylation is a definitive chemical process in which a methyl group is added to DNA. It usually occurs when a cytosine (C) is linked to guanine (G) through the phosphate (p) linkage resulting in a CpG site. The methylation reaction is catalyzed by a large family of DNA-methyltransferase (DNMTs) and distinct enzymes perform *de novo* DNA methylation and maintenance DNA methylation (Kotkar and Giri, 2020).

The epigenetic modifications can be inherited via mitotic and meiotic cell divisions and act to synchronize the changes in gene expression, which form the basis of memory responses (Eichten et al., 2014; Kinoshita and Seki, 2014; Avramova, 2015; Crisp et al., 2016; Liu and Avramova, 2016). Initial indications for involvement of chromatin modification in priming response came from studies in which plants primed with benzothiadiazole showed increased levels of histone methylation (Jaskiewicz et al., 2011). The bi- and tri-methylation of lysine 4 in histone 3 (H3K4me2 and H3K4me3, respectively) is correlated with active transcription of genes (Ruthenburg et al., 2007; Jaskiewicz et al., 2011) and were recognized as potential abiotic stress memory markers.

Response to HTS comprises of two components, induction of stress memory-associated genes and differential expression of HTS regulated genes. Both components are related with increased levels of H3K4me3 and H3K4me2 and are dependent on functional TFs (Lämke et al., 2016; Liu Y. et al., 2019). An increase in H3K4me2 and H3K4me3 was observed after heat priming in Arabidopsis (Conrath et al., 2015). H3K4me2/3 accumulation was also seen in Arabidopsis plants that had been primed with JA (Liu and Avramova, 2016).

Stress related memory might particularly develop when the plant is recovering from stress. This has been best exemplified by studies on vernalization leading to development of memory for cold stress. During the cold phase, repressive chromatin marks were stored at the nucleation regions of FLC, but epigenetic modifications were triggered after reversing to warm conditions. During the recovery from cold signal, the Polycomb Repressive Complex 2 (PHD-PRC2) was deregulated across the entire FLC locus and H3K27me3 increased remarkably throughout the entire gene to achieve epigenetic silencing (De Lucia et al., 2008; Angel et al., 2011).

Modification of chromatin provided potential memory for stress during priming events by maintaining the basal transcriptional machinery at the promoters of these genes to facilitate their rapid or strong expression in response to recurring stress (Conrath et al., 2015). Increase in H3K4me3 was retained as a memory on the stress responsive genes during stress, while H3K4me3 accumulated at primed defense genes before their transcription (Conrath, 2011; Jaskiewicz et al., 2011). Repeated exposures of Arabidopsis plants to mild drought like conditions increased the levels of H3K4me3 and stalled RNA polymerase II at RD29B and RAB18 loci allowing their rapid activation during the stressed phase (Ding et al., 2012). Increased H3K4me3 levels also caused increase in the occupancy of TATA-binding protein (TBP), a key step in the formation of the pre-initiation complex (PIC), at the promoters of memory genes (Liu and Avramova, 2016). HTS memory was also maintained by H3K27me demethylases, Relative of Early Flowering 6 (REF6) and the Jumonji (JMJ30) proteins by controlling the level of histone modification of *HSP22* and *HSP17*.6 genes (Yamaguchi and Ito, 2021).

Some stress-induced TFs were reported to function as prime components of transcriptional memory and are probably involved in recruiting specific chromatin-regulatory proteins to the target loci. For example, bZIP28/60 TFs were stimulated by the stress induced Unfolded Protein Response (UPR) protein (Liu and Howell, 2010) to recruit the COMPASS-like complex (Jiang et al., 2011) for initiating the accumulation of H3K4me during HTS (Song et al., 2015). It was shown that the same mechanism triggered the deposition of H3K4me3 at loci of dehydration memory genes. In yeast, both COMPASS and Mediator complexes were required for the H3K4me accumulation and transcriptional memory of the gene encoding inositol-1-phosphate synthase (Light et al., 2010, 2013; D’Urso et al., 2016). The composition of these protein complexes changed under memory and non-memory situations to allow differential regulation of transcription (D’Urso et al., 2016).

HSFA2 and HSFA1 are known to function in somatic HTS memory (Charng et al., 2007; Lämke et al., 2016; Liu Y. et al., 2019). Thermo-priming induced accumulation of H3K4me2 and H3K4me3 to generate HTS memory was dependent on HSFA2 (Lämke et al., 2016). Binding of HSFA2 occurred during first few hours of heat shock but the enhanced H3K4me3 and H3K4me2 levels persisted even after HSFA2 association with the gene had declined. The HSFA2 acts in trimeric complexes along with other HSFs like HSFA3 [reviewed in Scharf et al. (2012)], indicating that heat memory might be specific to few TF complexes and not individual TFs. HSEs were found to be the sites of active chromatin regions associated with histone (H3K9 and H3K14) acetylation and H3K4me3 (Guertin and Lis, 2010). Transcriptional activation of few HSF and HSP genes in Arabidopsis by HSF1A/B occurred through deposition of H3K56 acetylation under HTS (Weng et al., 2014).

The *trans*-generational HTS memory involves a heritable positive feedback loop consisting of REF6 and HSFA2 (Liu J. et al., 2019). The HSFA2 and REF6 promote the expression of each other to continuously maintain the active state of HSFA2. The HSFA2 in turn activates the expression of suppressor of gene silencing 3 (sgs3) interacting protein 1 (sgip1), an E3 ligase, to mediate SGS3 degradation. This represses biosynthesis of trans-acting siRNA (tasiRNA) and results in the release of its target, *Heat-Induced Tas1 Target 5* (*htt5*) to promote early flowering in the progeny plants. Subsequent studies demonstrated the role of FGT3 and HSFA3 in inducing transcription of memory-related genes during recovery from HTS.

Nucleosome remodeling has also been related to HTS induced memory. Arabidopsis FGT1 protein maintains low nucleosome occupancy at thermo-memory related genes like *hsa32, hsp22.0, hsp18.2, and hsp101*. FGT1 acts by interacting with proteins belonging to the chromatin remodeling complexes such as ATP-dependent Switch/Sucrose Non Fermentable (SWI/SNF), Imitation Switch family (ISWI) or Brahma (BRM), Chromo-domain Helicase DNA-binding (CHD) and INO80 complex ATPase subunit (INO80) to provide thermo-memory response (Brzezinka et al., 2016; Liu H. et al., 2021).

The *trans*-generational priming responses include both inheritable (DNA and chromatin structure change) and non-inheritable (metabolites, proteins or mRNA in the seeds) components (Boyko and Kovalchuk, 2011). The enzyme lysine-specific histone demethylase 1, which is involved in histone demethylation and epigenetic modification, was up regulated in the progenies of the primed plants. This suggested that the epigenetic modification could be involved in the *trans*-generational stress memory resulting in enhanced tolerance to stress.

The study of mechanism behind maintenance of epigenetic modifications over a longer period of time led to the discovery of trans factors that mediated faithful copying of the chromatin conformation through replication. One such protein, BRU, is necessary for the sustained activation of HTS memory genes (Suzuki et al., 2004; Takeda et al., 2004; Brzezinka et al., 2019). It is also essential to sustain the transcription of HTS responsive genes *hsa32*, *apx2*, *hsp22.0*, and *hsp21* (Stief et al., 2014). BRU1 has been implicated in the inheritance of chromatin states in transcriptional silencing and the DNA damage response across DNA replication and cell division.

Recently, a BRU1 orthologue from mammals was found to bind to single-stranded DNA and newly incorporated nucleosomes after replication (Huang et al., 2018). The histone chaperone, Chromatin Assembly Factor-1 (CAF-1) plays a key role in depositing histone H3H4 tetramers into newly replicated DNA. Mutants of *caf-1* showed a constitutive priming response against pathogens and increased H3K4me3 at primed defense-response genes. This suggested that regulated deposition of histone tetramers may be essential for inheritance of priming responses (Mozgova et al., 2015).

DNA methylation is another mechanism of epigenetic modification and is shown to be a dynamic regulatory mechanism of defense genes and stress priming. Single base-resolution DNA methylome profiling of cold primed tissues revealed the global loss of DNA methylation with increase in some locally hypermethylated sites. The *de novo* methylation of cytosine residues (CG, CxG, Cxx) in plant DNA can be triggered by small interfering RNAs (siRNA). The onset of RNA-directed DNA methylation begins with the formation of siRNAs. The siRNAs may be produced through the Polymerase IV (Pol IV) pathway and loaded in Argonaute 4 (AGO4) complex. Alternatively, the siRNAs can be generated through Pol I-RNA Dependent RNA Polymerase 6 (RDR6) pathway and are loaded into AGO6 complex. The AGO4/6 complex, interact with Pol V to recruit a Nuclear RNA Polymerase E1 (NRPE1) complex, which subsequently establishes DNA methylation through Domains Rearranged Methyltransferase2 (DRM2). During cell divisions, the maintenance of known DNA methylation marks in CG and CxG are catalyzed by Methyltransferase1 (MET1) and Chromomethylase3 (CMT3), respectively (Ramirez-Prado et al., 2018; Zhang H. et al., 2018).

## Role of Phytohormones in Stress Priming

The classic growth promoting phytohormones viz. auxin, cytokinin (CK), strigolactones (SLs) and BR and the stress related hormones such as ABA, JA and SA, play an essential role in orchestrating protection in response high temperature and other abiotic stresses (Tang et al., 2008; Verma et al., 2016; Kumar et al., 2019; Li et al., 2021). Phytohormones act by positively regulating stress responsive gene expression, nutrient allocation, photosynthetic activity, osmolyte biosynthesis and antioxidant metabolism (Alam et al., 2013; Peng et al., 2014; Qiu et al., 2014; Shi et al., 2014; Jin and Pei, 2015; Cheng et al., 2016). Exogenous application of ABA, ethylene and SA or enhancing their endogenous levels helped to significantly overcome HTS induced damage and improved thermo-tolerance response (Table 2). Combined application of gibberellins and ABA in rice lead to increase in pollen germination and vigor. The phytohormones acted by improving anti-oxidant activity and membrane stability (Chhabra et al., 2009; Song et al., 2012). The role of ethylene in reducing HTS by reducing oxidative damage has been studied at seedling stage in Arabidopsis and rice (Larkindale and Knight, 2002; Wu and Yang, 2019).

**TABLE 2:** Role of hormones in priming plants to high temperature stress.

Elicitor	Priming treatment			Subsequent stress		Effect of priming	References
	Stage	Concentration	Plant	Stress	Duration		
Cytokinin	3 leaf seedling	BAP (60 mg L^–1^)	*Oryza sativa*	31.5 and 38.3°C*	15 days	Cytokinin transport from root to shoot for determining panicle size	Wu et al., 2017
	Anthesis	10 mg L^–1^ (6-BA)	*Triticum aestivum*	35/20°C*	5 days	Significantly (*P* < 0.05) enhanced the endosperm cell division, grain-filling and 1,000-grain weight	Yang D. et al., 2016
	Seedling	10 and 100 μM	*Agrostis stolonifera*	38°C/28°C*	4 weeks	Provides protection via antioxidants and shielding the photosynthetic apparatus	Wang et al., 2013
	Seedling	50 μM, 100 μM, 250 μM, and 500 μM	*Brassica juncea*	47.5°C	-	Soaking seeds in 50 and 100uM kinetin are effective in mitigating HTS.	Chhabra et al., 2009
	5 week plants	-	*Arabidopsis thaliana*	45°C	3 h	Enhanced activity of NADPH oxidases (NOX) and antioxidant enzymes [superoxide dismutases, guaiacol peroxidases, catalases, ascorbate peroxidases]	Prerostova et al., 2020
Gibberlic acid -3 (GA3)	3 months seedlings	100 μM	*Phoenix dactylifera*	44°C	6 weeks	Accumulation of polyphenol oxidase, peroxidase, and ascorbate peroxidase activities and up regulation of their synthesis, activation of HSF related genes (especially *hsfA3*)	Khan et al., 2020
	Seedling	50 μM, 100 μM, 250 μM, and 500 μM	*Brassica juncea*	47.5°C	-	Soaking seeds in GA was effective in mitigating HTS	Chhabra et al., 2009
	Post anthesis	100 μM in culture media	*Triticum aestivum*	45°C	2 h daily for 5 days	Positively regulates grain sink activity, sucrolytic and aminotransferases	Asthir and Bhatia, 2014
	Seedling stage	50 mM	*Arabidopsis thaliana*	50°C	3 h	Counter balances the inhibitory effects of stress during seed germination and seedling growth by modulation of SA biosynthesis	Alonso-Ramírez et al., 2009
Auxin	Anthesis	1, 10, 50 and 100 μM L^–1^ (NAA)	*Oryza sativa*	40°C	2 h	Prevents the inhibition of pollen tube elongation in pistil and crosstalk with ROS	Zhang C. et al., 2018
	5-leaf seedlings	10^–6^, 10^–5^ or 10^–4^ M	*Hordeum vulgare*	31°C	5 days	Restores DNA replication licensing factor MCM5 expression and helped in normal proliferation and development of anther cells	Sakata et al., 2010
	Seedling stage	50 μM, 100 μM, 250 μM, and 500 μM	*Brassica juncea*	47.5°C	-	IAA is effective in mitigating HTS	Chhabra et al., 2009
ABA	5–6 leaf seedlings	1 μM L^–1^, 10 μM L^–1^, and 100 μM L^–1^	*Oryza sativa*	45°C	24 h	Maintenance of energy homeostasis	Li et al., 2020
	Seedlings	1, 10, 100 μM L^–1^		39–41°C	7 days	ABA prevents the decrease in pollen viability and spikelet fertility	Rezaul et al., 2019
	30 days old plants	30 μM	*Arabidopsis thaliana*	38°C	8 h	Induces accumulation of APX1 and MBF1c	Zandalinas et al., 2016
	2 months old plants	10 μM	*Festuca arundinacea*	38/33°C*	25 days	Up regulation of CDPK3, MPK3, DREB2A, AREB3, MYB2, MYC4, HsfA2, HSP18, and HSP70 for maintaining transcription	Zhang et al., 2019a
	4 weeks seedlings	5 μM		37/32°C*	35 days	Increased expression of HSFA2c, HSPs and ABA-responsive transcriptional factors, increased leaf photochemical efficiency and membrane stability	Wang X. et al., 2017
	4 days seedlings	2.5 μM	*Cicer arietinum*	30/20, 35/25, 40/30, and 45/35°C*	10 days	Reduced MDA and H_2_O_2_ concentrations and upregulated HSPs	Kumar et al., 2012
Ethylene	12 days old seedlings	10 μM ACC	*Oryza sativa*	45°C	4 days	Reduced electrolyte leakage and MDA Increased enzymatic activity of CAT, APX, and POX	Wu and Yang, 2019
	Tomato plant	0.1 μL L^–1^ in air	*Solanum lycopersicum*	45°C	2 h	Ethylene synthesis genes SlACS3 and SlACS11 are increased	Jegadeesan et al., 2018
Salicylic acid	5 weeks old plants	0.5 mM NaSA	*Brachypodium distachyon*	35°C	4 h	Induction of chlorophyll-a and changes in various protective compounds, such as glutathione, flavonoids and antioxidant enzymes	Janda et al., 2020
	4-day-old seedlings	10 and 20 μM	*Brassica*	55°C	3 h	Help through enhancing seedling length, HSP expression, total soluble sugars and enzymatic activities of invertase, CAT, PO. Reduce electrolyte leakage and confer membrane protection thereby acquiring thermo-tolerance.	Kaur et al., 2009
	Fully expanded fifth leaf	1 mM	*Solanum lycopersicum*	42°C	36 h	SA enhanced gaseous exchange parameters, water use efficiency, decreased electrolyte leakage and increased SOD and improved thermotolerance.	Jahan et al., 2019
	Seven day seedlings	0.5 mM and 1 mM for 3 h	*Cajanus cajan*	45°C	3 h	Enhanced Antioxidant defense system.	Kaur et al., 2019
	Sowing and reproductive stages	50 ppm	*Gossypium hirsutum*	45/30°C, 38/24°C, 32/20°C*	7 days	Enhanced CAT, SOD activity, net photosynthetic rate chlorophyll content, number of sympodial branches, boll weight, and fiber quality	Sarwar et al., 2018
	Seedling	100 μL of 1 mM	*Pisum sativum*	28/24°C*	16 h	Enhanced net CO_2_ assimilation and chlorophyll content	Martel and Qaderi, 2016
	pollen mother cell	0.01, 0.1, 1.0, 10, and 50 mM	*Oryza sativa*	40°C	10 days	H_2_O_2_ may play an important role in mediating SA to inhibit pollen abortion caused by heat stress by hampering the tapetum PCD.	Feng et al., 2018
	10 day seedlings	10 mM	*Triticum aestivum*	38°C	2 h	Enhanced total antioxidant capacity, accumulation of SAGs and osmolytes	Kumar et al., 2015
Brassinolide	16–18 cm high plant	0.01, 0.1, and 1.0 mgL^–1^	*Leymus chinensis*	38/25°C*	10 h	increased biosynthesis of photosynthetic pigments, osmolytes, antioxidant enzyme and thus thermotolerance	Niu et al., 2016
	3 weeks old seedlings	10^–6^M	*Brassica napus*	45°C	4 and 8 h	Enhanced endogenous ABA content	Kurepin et al., 2008
24-epibrassinolide	3–4 leaf seedling stage	0.05, 0.1, 0.5, 1.0, and 1.5 mg dm^–3^	*Cucumis melo*	42/32°C*	2 days	Reduced MDA content and enhanced content of soluble proteins, free proline and antioxidant enzymes including guaiacol peroxidase, catalase, superoxide dismutase and ascorbate peroxidase	Zhang Y. et al., 2014
	12 days old seedlings	0.005 and 0.25 mg dm^–3^	*Hordeum vulgare*	42°C	3 h	Increased the PSII efficiency	Janeczko et al., 2011
	20, 22, or 24 days after sowing	0.01 μM	*Triticum aestivum*	35/28 or 40/35°C*	24 h	Improved the growth features, photosynthetic efficacy and various biochemical parameters through enhanced antioxidant system and osmoprotectants	Hussain et al., 2019
Jasmonic acid	3 week plants	5 μm	*Arabidopsis thaliana*	45°C	2 h	Modulating ethylene levels. Ethylene mutant *ein2-1* conferred greater thermo-tolerance	Clarke et al., 2009
	Anthesis	50, 100, 150 μM JA and 50, 100, 150 μM MeJA,	*Oryza sativa*	25°C–35°C	6 days	Increase in soluble sugars, activities of catalase and α-amylase and reduction in H_2_O_2_ increased rate of spikelet opening	Yang et al., 2020
	37 days plants	20, 40, 60, 80, 100, 150, and 200 μM L^–1^	*Lolium perenne*	38/30°C*	14 days	MeJA-induced heat tolerance by maintenance of chlorophyll loss, photosynthesis relative water content (RWC), electrolyte leakage (EL) and malondialdehyde (MDA) content under HTS	Su et al., 2021
Stringolactones	4 week plants	0.01 μM GR24	*Festuca arundinacea*	35/30°C*	8 days	Leaf elongation due to up regulation of cell-cycle-related genes and down regulation of auxin transport related genes	Hu et al., 2019
	14 days seedlings	10 μM rac-GR24	*Lupinus angustifolius*	40°C	1 h	Increased proline content and activities of antioxidant enzyme, reduced lipid peroxidation	Omoarelojie et al., 2020

*indicates day/night temperatures.

### Abscisic Acid

The primary stress hormone, ABA, modulates plant responses to multifarious environmental stresses. The involvement of ABA in response to HTS and thermo-priming has emerged through many different studies (Larkindale and Knight, 2002; Zhou et al., 2014; Li et al., 2021). ABA-mediated ROS accumulation in guard cells caused closure of stomata (Kwak et al., 2003) and helped to decrease loss of water. This resulted in cross-tolerance to temperature (heat or cold) and drought stresses. The ABA signals activated the expression of several stress responsive genes such as *dehydration-responsive 22* (*rd22*), *pr1a.205, thaumatin-like protein 4* (*tlp4*), and *myb* in wheat resulting in increased drought tolerance and fungus resistance (Zhang et al., 2012). ABA enhanced tolerance to HTS by up regulation of HSFs and HSPs, increase in sugar metabolism and reduction in electrolyte leakage (Larkindale and Knight, 2002; Wind et al., 2010; Wang X. et al., 2017; Rezaul et al., 2019). Arabidopsis plants treated with inhibitor of ABA biosynthesis showed impaired response to HTS and reduction in ROS levels (Larkindale et al., 2005). HTS also evoked a rapid and transient expression of endogenous ABA that in turn increased the levels of ROS like H_2_O_2_ thereby augmenting thermo-tolerance (Larkindale et al., 2005).

Interaction of ABA and ROS is considered as one of the prime factors for the acclimation of plants exposed simultaneously to drought and salt stresses (Suzuki et al., 2016; Zandalinas et al., 2016). Mutants in ABA biosynthesis or signaling components displayed suppressed H_2_O_2_ accumulation and increased HTS sensitivity in plants (Larkindale et al., 2005). Loss-of-function mutation in the ABA induced NADPH oxidases, also known as RBOHs exhibited impaired HTS tolerance as measured by decrease in seed survival and germination capacities (Larkindale et al., 2005; Silva-Correia et al., 2014). Heat- or cold- priming were also found to increase the endogenous concentration of ABA, SA and H_2_O_2_ (Liu et al., 2006; Wan et al., 2009). Cold priming induced frost tolerance in winter and spring wheat (*Triticum monococcum*) was associated with greater accumulation of ABA and dehydrins (Vanková et al., 2014). Priming with drought induced cold stress tolerance was associated with ABA and ROS accumulations in the leaf tissues of wheat (Li X. et al., 2015).

### Jasmonic Acid and Salicylic Acid

The biotic stress-induced hormones, JA and SA, can prime transcription by stimulating the expression of defense genes after attack by the pests or pathogens (Beckers and Conrath, 2007; Chen R. et al., 2012). The basic helix–loop–helix (bHLH) TF, MYC2, is an important part of the JA signaling pathway and its activity is regulated by binding of repressor (JAZs) and co-repressors (TPL or related TRPs) proteins (Chini et al., 2007). The JA-induced proteolytic degradation of JAZ repressors allows MYC2 to bind with promoters. It then interacts with MED25 subunit to facilitate the assembly of Mediator complex (Elfving et al., 2011; Çevik et al., 2012), which in turn recruits the pre-initiation complex (PIC) to activate transcription. The cross-talk between JA- and ABA-mediated pathways, has been well established (Wasternack and Hause, 2013; de Ollas et al., 2015). Both JA and ABA mediate induction of drought-inducible genes in response to dehydration stress at the initial stage. However, in subsequent dehydration stress, ABA produced during the initial stress reduces the production of the MYC2 to inhibit biosynthesis of JA (Avramova, 2019).

The role of SA has been discussed in numerous HTS related studies in plants. Exogenous application of SA or its functional synthetic analogs like BTH or INA (2,6-dichloroisonicotinic acid) and the non-protein amino acid, BABA (b-amino butyric acid), prior to HTS were shown to enhance plant biomass, height and photosynthetic efficiency (Kauss et al., 1992; Oostendorp et al., 2001). Priming of plants with BABA also induced SA signals and activated the defense response (Ton et al., 2005). SA enhanced the activities of antioxidant enzymes like SOD, CAT and peroxidases to scavenge ROS and increased enzyme activities required for proline biosynthesis as an adaptive strategy to tolerate HTS. It also helped in reducing membrane damage caused by HTS and decreased the proline-metabolizing enzymes (Verbruggen and Hermans, 2008; Szabados and Savouré, 2010; Lv et al., 2011). Application of SA at seedling stage in plants such as maize, potato, wheat, cotton, pigeon pea, Arabidopsis, mung bean, grape and alfalfa lead to increase in antioxidant enzymes, photosynthetic activity and proline accumulation which reduced cellular damage. It also enhanced basal thermo-tolerance by up regulating HSP expression in some cases (Clarke et al., 2004; Saleh et al., 2007; Khan et al., 2013; Wang et al., 2014a; Hameed and Ali, 2016; Khanna et al., 2016; Makarova et al., 2018; Jahan et al., 2019; Kaur et al., 2019; Wassie et al., 2020).

The Set Domain Group 8 (SDG8) proteins which represent the major H3K36 di- and tri- methyltransferase in Arabidopsis have been implicated in the JA- and SA-mediated plant defense (Ding and Wang, 2015; Li Y. et al., 2015). SDG8 is essential for H3K36me3 for expression of the *R*-gene, *lazarus 5* (*LAZ5*), in response to fungal infection (Berr et al., 2010; Palma et al., 2010). This memory can be transmitted to the progeny plants and the off-springs were primed for enhanced activation of the WRKY6, PR1 and WRKY53 genes that play roles in immunity (Slaughter et al., 2012).

### Auxin

Warm ambient temperature signals can promote a series of structural changes in plants like elongation, growth and flowering, which are collectively termed as thermo-morphogenesis (Quint et al., 2016) and auxin plays a major role in these processes. At normal growth temperatures, the exogenous application of auxin does not elicit hypocotyl elongation but at higher temperatures the auxin-responsive gene expression causes hypocotyl elongation and leaf hyponasty (Küpers et al., 2020). Auxin also act as thermo-protectant of anthers in barley and rice (Sakata et al., 2010; Oshino et al., 2011; Zhang C. et al., 2018).

The light responsive Phytochrome Interacting Factor 4 (PIF4) and PIF7 are involved in regulating hypocotyl elongation and leaf hyponasty. Recent studies showed that PIFs are inducible by HTS and they interact with at least one of the phytochromes thereby indicating their important role in transducing both light and temperature signals (Choi and Oh, 2016; Pham et al., 2018; Kim et al., 2020). The temperature-induced hypocotyl elongation is delayed by the suppression of PIF4 activity by blue light photoreceptor, Cryptochrome 1 (CRY1) (Ma et al., 2016). Loss-of-function mutants of *pif4* and *pif7* abrogate HTS mediated thermo-morphogenesis and show drastic reductions in auxin biosynthetic enzymes (aminotransferase, YUCCA and cytochrome P450s) (Sun et al., 2012; Fiorucci et al., 2020).

### Cytokinin

Several studies have suggested the role of CK in acquisition of thermo-tolerance by inducing higher antioxidant activity and metabolism. When a part of the plant or whole plants were exposed to HTS, a rapid increase in CK was observed which further activated the carbohydrate metabolism and photosynthetic genes (Dobrá et al., 2015). Agrobacterium mediated ectopic transcription of *isopentenyltransferase* (ipt), which encodes a key enzyme in CK biosynthesis system, resulted in plants that were better adapted to HTS (Skalák et al., 2016). An interesting pattern of expression of interconnected proteins was observed in the chloroplasts, in response to HTS and CK. The adverse effect of HTS on spikelet formation and panicle differentiation is mitigated by CK application (Wu et al., 2017). In Arabidopsis and in crops like maize, wheat and rice, CKs were shown to provide HTS tolerance by reduction in kernel abortion, increase in rate of grain filling, modulating spikelet injury and regulating ROS production (Cheikh and Jones, 1994; Yang D. et al., 2016; Wu et al., 2017; Prerostova et al., 2020).

### Brassinosteroids

Brassinosteroids are known to perform surveillance for HTS induced lipid peroxidation, ion leakage and survival rate (Mazorra et al., 2011). Treatment of plants with BR lead to a rise in basic thermo-tolerance due to higher translation of proteins like HSPs, aquaporins, etc. (Dhaubhadel et al., 2002; Sadura et al., 2020). The levels of ROS and expression of antioxidant enzymes also spiked up after the application of exogenous BR under HTS (Nie et al., 2013). However, in tomato seedlings both overproduction and deficiency of BR showed similar response to thermo-tolerance indicating that the process might be independent of BR homeostasis (Mazorra et al., 2011). Later it was shown that Brassinazole-resistant 1 (BZR1), the prime TF in BR signaling, activated PIF4 gene expression to integrate the plant response to different environmental stimuli (Ibañez et al., 2018). BRs also influenced the levels of other phytohormones like ABA, ethylene and SA to regulate oxidative stress under HTS and boost thermo-tolerance (Divi et al., 2010; Kothari and Lachowiec, 2021).

### Melatonin

The role of phyto-melatonin is also implicated in governing the response and memory to plant stress (Arnao and Hernández-Ruiz, 2019). Melatonin, a predominantly animal hormone, is present in plants and performs a number of physiological activities including chlorophyll preservation, root and shoot growth, photosynthesis, reduction of oxidative damage and suppression of leaf senescence (Byeon et al., 2012; Tan et al., 2012; Zhang N. et al., 2014; Li X. et al., 2018). Exogenous application of melatonin to roots resulted in its absorption and mobilization in xylem and subsequent accumulation in the leaves (Yoon et al., 2019). Extracellular melatonin enhanced the level of *TaSNAT* transcripts, which encode a key enzyme in the melatonin biosynthetic pathway and increased the intracellular melatonin levels.

Various reports have also shown the protective effects of phyto-melatonin against biotic and abiotic stress (Li et al., 2012; Li X. et al., 2018; Bajwa et al., 2014; Meng et al., 2014; Shi et al., 2015b). Melatonin is an important master regulator of redox homeostasis in plants (Arnao and Hernández-Ruiz, 2019). It induced the DREB1/CBF TFs AtCBF1, AtCBF2, and AtCBF3 that caused increased tolerance to drought, cold, salt, and Pst-DC3000 (Shi et al., 2015a). Melatonin also up regulated polyamine levels by promoting the synthesis of polyamines from its precursor amino acids, arginine and methionine. In wheat seedlings, Melatonin attenuated the effect of salt stress by preventing polyamine degradation (Ke et al., 2018).

## Conclusion and Perspectives

According to Intergovernmental Panel on Climate Change (IPCC, 2018), the unprecedented increase in global mean temperatures has caused an upsurge in the incidence of extreme HTS. Its adverse effects on plant growth, reproduction and yield are proportional to the intensity and duration of high temperatures (Prasad et al., 2017; Ali et al., 2020). The increase in global temperatures accompanied by fluctuations or changes in climate regimes has increased the occurrence of abiotic stresses such as HTS, salinity, drought and flooding as well as biotic stresses such as pathogen infections and pest invasions. These challenges have emerged as major factors limiting crop production yield (Pandey et al., 2017).

High temperature stress results in various impairments including early leaf senescence, accumulation of ROS, reduced germination, decreased rate of photosynthesis, inhibition of root growth, lower seed set, reduced vigor and elevated rates of lipid peroxidation, protein denaturation, spikelet sterility and anther indehiscence (Wahid et al., 2007; Hasanuzzaman et al., 2013; Rodríguez et al., 2015; Narayanan et al., 2016; Iqbal et al., 2017; Ohama et al., 2017; Ali et al., 2020). Sustainable agriculture necessitates the adaptation of strategies to boost plant responses to improve their tolerance to these stresses. Although lot of efforts like conventional breeding, application phytohormones, comparative genetics, etc., have been initiated in this direction but a detailed understanding of the underlying mechanisms is still needed.

Priming has the potential to be developed as a very promising technique for improving the long-term sustainability of agriculture and increasing crop yields in the present context of global warming and climate change. Plants can be thermo-primed by moderately high temperatures to sustain extreme temperatures that are otherwise fatal to an unadapted plant (Bäurle, 2016). In our lab, experiments on thermo-priming mature rice before and after flowering, showed that priming allows the crops to tolerate HTS and help in prevention of seed loss and increase seed production by sustaining grain filling (Kushawaha et al., 2021). The response may vary from crop to crop depending upon their life cycle, habitat and stress tolerance levels. Therefore comprehensive investigations are required using a broad range of plant species to determine the most efficient and effective stage and duration of priming. The analyses also need to be extended from study of individual stresses to include a mixture of stressors so that plants can be acclimated to tolerate a wide range of temperatures.

Epigenetic regulations, transcript modifications, changes in protein conformations and generation of hormonal or metabolic signals are only a few of the cellular processes that have been studied in response to stress priming in the last decade. Epigenetic alterations resulting in changes in structure and composition of chromatin may play a vital role in stress memory by regulating transcription and/or plant response. The mechanisms overlap with the stress response pathways and often involve activation of epigenetic imprints and feedback loops to initiate faster recruitment of the stress response.

Advance investigations at the chromatin level will be fundamental for understanding the nature of modifications involved in inheriting tolerance to different stresses in the same species. Few features like accumulation of H3K4me may be commonly related to more than one type of stresses, while memory of individual stress may involve specific molecular elements. More research is required to recognize these patterns of stress memory and determine the exact mechanisms by which they are generated. The importance of synchronized gene silencing by miRNAs and other regulatory non-coding RNAs in stress primed plants is a significant question which will require an in-depth analysis. Their role and pathways for integration of priming with stress memory in regulation of plant development and productivity will be an important question to investigate.

It is apparent that our understanding of effects of priming and induced memory of stress in plants is far from clear. The maintenance of memory and its inheritance across cell divisions needs to be investigated. It will be essential to investigate how long the memory phase is maintained and regulated. Further studies are also required to comprehend the precise mechanisms that elicit and reset stress memory. The advancements in various omic-technologies will help in providing better understanding of priming induced cross-talk during stress tolerance in plants. It is evident that increased knowledge of the priming pathways will aid in designing strategies for improving plant performance and seed production. This will not only help them to pre-adapt to changing environmental conditions but also enable them to habituate in new domiciles.

## Author Contributions

NS-M and SKS conceptualized the idea of the study. AK, VK, and KP prepared the draft. SKS and NS-M edited the manuscript. All authors read and approved the final manuscript.

## Conflict of Interest

The authors declare that the research was conducted in the absence of any commercial or financial relationships that could be construed as a potential conflict of interest.

## Publisher’s Note

All claims expressed in this article are solely those of the authors and do not necessarily represent those of their affiliated organizations, or those of the publisher, the editors and the reviewers. Any product that may be evaluated in this article, or claim that may be made by its manufacturer, is not guaranteed or endorsed by the publisher.
